# Immunocapture strategies in translational proteomics

**DOI:** 10.1586/14789450.2016.1111141

**Published:** 2015-11-11

**Authors:** Claudia Fredolini, Sanna Byström, Elisa Pin, Fredrik Edfors, Davide Tamburro, Maria Jesus Iglesias, Anna Häggmark, Mun-Gwan Hong, Mathias Uhlen, Peter Nilsson, Jochen M Schwenk

**Affiliations:** ^a^ Affinity Proteomics, SciLifeLab, School of Biotechnology, KTH - Royal Institute of Technology, Solna, Sweden; ^b^ Department of Oncology-Pathology, Clinical Proteomics Mass Spectrometry, SciLifeLab, Karolinska Institutet, Solna, Sweden

**Keywords:** Antibodies, immunocapture, mass spectrometry, protein enrichment, sandwich assays, SRM

## Abstract

Aiming at clinical studies of human diseases, antibody-assisted assays have been applied to biomarker discovery and toward a streamlined translation from patient profiling to assays supporting personalized treatments. In recent years, integrated strategies to couple and combine antibodies with mass spectrometry-based proteomic efforts have emerged, allowing for novel possibilities in basic and clinical research. Described in this review are some of the field’s current and emerging immunocapture approaches from an affinity proteomics perspective. Discussed are some of their advantages, pitfalls and opportunities for the next phase in clinical and translational proteomics.

Antibodies are one of the most fascinating and versatile proteins found in vertebrates. Produced by the immune system to recognize pathogens and trigger their neutralization, the antibodies present in any individual are unique in their specificity and comprised a large repertoire generated through an elaborate process of random genetic recombination and affinity selection.[1] Since late in the 18th century, circulating antibodies have been investigated as biomarkers [1–4] and seen as natural amplifiers of traces of diseases, such as infections or pathologies causing aberrant protein secretion, expression or modification.[5,6] Nowadays, antibodies are produced through animals’ immune system or recombinant as reagents for many biotechnological and clinical applications. Immunoassays such as enzyme-linked immunosorbent assay (ELISA), immunohistochemistry (IHC) and flow cytometer assays are among those routinely found in diagnostic laboratories. For example, IHC is used to detect protein markers in tissue biopsies supporting cancer diagnosis, prognosis and monitoring, subclassification of neurodegenerative disorders and diagnosis of muscular diseases [7,8]; serum biomarkers such as prostate-specific antigen and cancer antigen 125 are determined via sandwich ELISA for monitoring prostate, pancreatic and ovarian cancer [9]; and flow cytometer assays are employed for phenotyping, diagnosis and staging of hematological neoplasm, for monitoring purposes in chemotherapy, and HIV infection through count of CD4 positive lymphocytes.[10] Several formats of multiplex immunoassays have been developed and primarily applied for protein profiling, quantification and functional studies in preclinical research using tissues, cell lysates or body fluids.[4,11–14] Clinical applications of commercialized protein arrays for diagnosis purposes are still limited to autoimmune and infectious disease,[15] nevertheless they show a potential in preclinical research also to be translated into personalized medicine applications. The ability of antibodies to capture a target protein with high affinity and specificity has been exploited for enrichment and quantification of proteins and peptides in integrated proteomic assays which use mass spectrometry (MS) as a final read-out.[16,17]

The aim of this review is to provide a survey of some of the currently used immunocapture (IC) assays and technologies focusing on recent achievements. We will introduce how MS-based detection in combination with antibody applications can accelerate translational proteomics. The review will provide a perspective on how traditional and novel assays may lead to a better understanding of human biology, support clinical diagnosis and treatment of human diseases. The different strategies discussed are summarized in Figure 1 and their analytical features are compared in Table 1.

**Figure 1.**
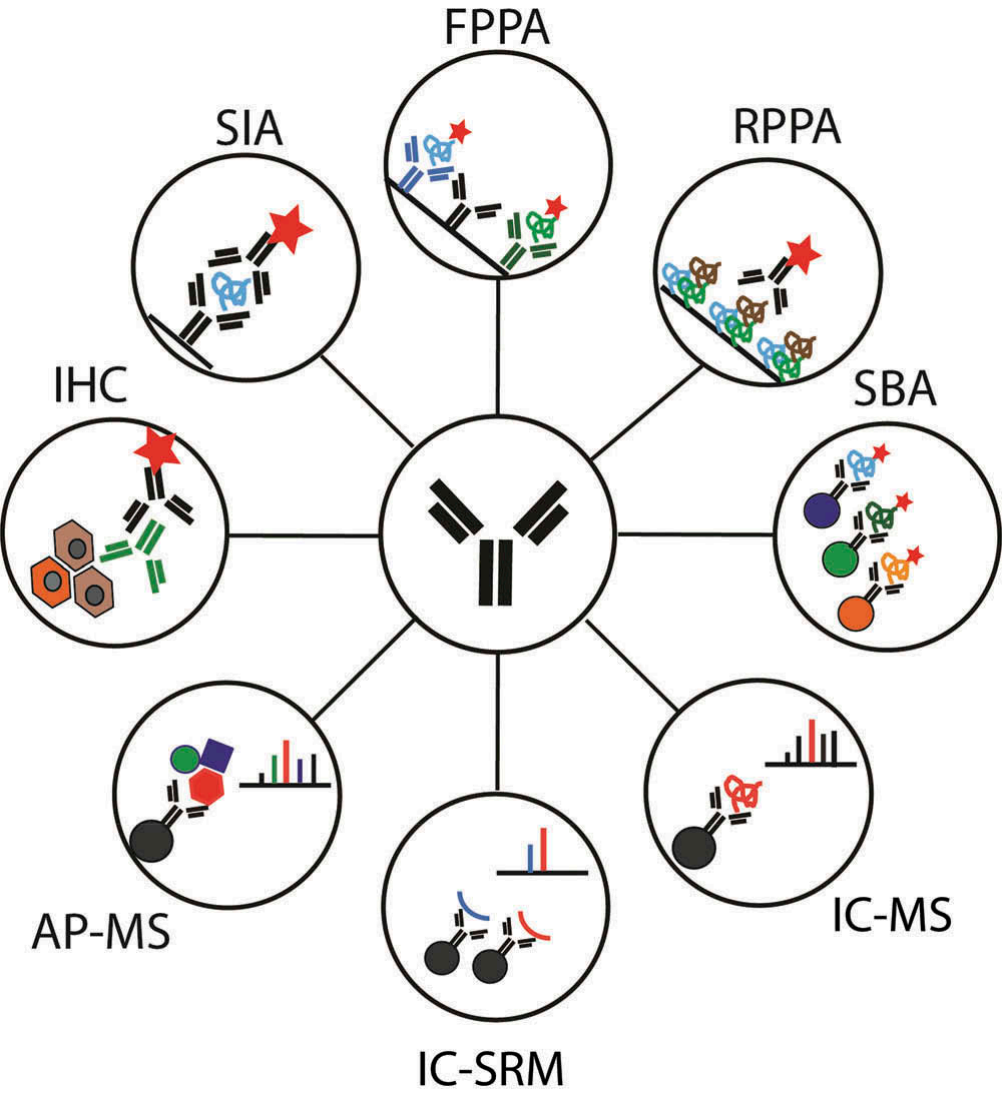
Schematic representation of immunocapture strategies discussed in this review. **AP-MS: Affinity purification mass spectrometry for interaction studies; FPPA: Forward phase protein array; IC-MS: Immunocapture coupled to mass spectrometry (DDA or DIA); IC-SRM: Immunocapture coupled to SRM; IHC: Immunohistochemistry; RPPA: Reverse phase protein array; SBA: Suspension bead arrays; SIA: Sandwich immunoassay.**

**Table 1.** Main analytical features of antibody capture technologies.

	SIA	**TMA-IHC**^†^	FPPA	RPPA	SBA	IC-MS	IC-SRM
Specificity	High	Low	Low	Low	Low	High	High
**Sensitivity**	pg/ml	Semiquantitative^‡^	ng/ml	ng/ml	ng-pg/ml	ng-pg/ml	ng-pg/ml
**Throughput** **(samples)**	~96	~230	~12	~500	~384	~5 (same time of an ELISA)	~40
**Multiplex** **(affinity reagents)**	Low	Low	High	Low	High	High	High
**Automation**	High	Low	Low	Low	High	High	High
**Turnaround time**	Low	Low	High	High	High	High	High
**Sample type**	Fluid	Tissue	Tissue	Fluid/tissue	Fluid/tissue	Fluid/tissue	Fluid/tissue
**Sample required**	1–100 µl		50 µg	0.1 µl/10 ng	0.1 µl/50 µg	10–1000 µl/1–50 mg	10–1000 µl/1–50 mg
**Applications**	Clinical	Clinical	Discovery/clinical	Discovery/clinical	Discovery	Validation	Validation

^†^High-throughput TMA-IHC. ^‡^For TMA-IHC, it is not possible to provide an absolute concentration, but continuous values of protein expression in tissues are obtained by methods such as Automated Quantitative Analysis (AQUA).[8] For this reason, the method has been defined semi-quantitative. FPPA: Forward phase protein array; IC-MS: Immunocapture coupled to mass spectrometry (shotgun); IC-SRM: Immunocapture coupled to mass spectrometry (selected reaction monitoring); IHC: Immunohistochemistry; RPPA: Reverse phase protein array; SIA: Sandwich immunoassay; SBA: Suspension bead arrays; TMA-IHC: Tissue microarray immunohistochemistry.

## 1. Potentials and challenges of affinity-based proteomics

### 1.1 Affinity reagents and their resources

Antibodies are still the most commonly used tools in affinity-based assays; unfortunately, a general problem exists to find the right antibodies for the application of interest. It has been estimated that of the over 2,000,000 commercially available antibodies, only 500,000 of these are unique. Several publications report that a low percentage of them actually show required performance in terms of specificity. Within the Human Protein Atlas (HPA) project, 20,000 commercial antibodies have been tested and only 50% were found to be effective in IHC staining.[18] Egelhofer and co-workers performed an evaluation of 246 antibodies in 2011 used in epigenetic studies targeting different histone modifications in *Drosophila melanogaster*, *Caenorhabditis elegans* and human cells. Overall, 25% failed specificity and functionality could not be demonstrated across different applications.[19] Several online catalogues have been created in order to provide a valuable resource of antibodies. Antibodypedia [20,21] is one such catalogue containing more than 1,800,000 antibodies covering 94% of the protein-encoding genes in humans.[21] Antibodypedia recently started an “Affinity Binder Knock-Down Initiative” to assure the performance of binding reagents in specific applications. Additional resources include antibodies-online,[22] which set up a program for antibody validation in independent labs, Linscott’s Directory,[23] the commercial guide Biocompare [24] and the nonprofit Antibody Registry.[25]

The Clinical Proteomic Tumor Analysis Consortium promotes the evaluation of antibodies in methods such as ELISA and Western blots to demonstrate the interplatform applicability of such reagents. Antibody [26] and Assay Portals [27] are the online resources related to Clinical Proteomic Tumor Analysis Consortium. Polyclonal antibodies have been widely used in research for decades, but discussion about their analytical specificity, batch-to-batch variability and limited ability to produce the desired quantities on demand and for future assays will in the long run lead to renewable binders taking their place. A big effort is required to produce and evaluate renewable affinity reagents, such as monoclonal antibodies from not only mice but also alterative antibody isoforms such as recombinant single-chain variable fragments. The latter have been exploited for proteomic profiling within various cancers and autoimmune diseases [28–30] while recombinant antibody fragments have also been used for peptide enrichment.[31] In addition, recombinant affinity reagents such as SOMAmers, which are short single-stranded oligonucleotides based on aptamer reagents,[32] were recently used to study Alzheimer’s disease (AD) [33] and muscular dystrophies.[34] There are other novel available affinity reagents used in immunoassays including nanobodies, camelids single-domain antibodies,[35,36] designed ankyrin repeat proteins [37] and affibody molecules.[38]

### 1.2 Mapping the human proteome by antibodies and MS

A deeper knowledge of the human proteome could lead to a better understanding of diseases and how to treat them. For many years, proteomics studies have provided us with a grainy picture of the complexity of biological functions behind physiological and pathological conditions. For this reason, an increasing effort has been made to generate a global map of the human proteome based on the identification of all the constituents of human protein-coding genes.[39–41] Such resources now provide a valuable basis to accelerate our knowledge of the proteome and serve both basic and clinical proteomic research.

One of these efforts is the HPA project, a unique initiative started in 2003 with the aim to produce antibodies to all human proteins. HPA aims to provide a map of the human proteome based on IHC and immunofluorescence imaging, and lately also RNA sequencing.[42] To date, more than 47,000 protein array–validated polyclonal antibodies have been produced using antigen sequences of 50–150 residues and more than 13 million of IHC images have been collected. At least one major isoform of the 85% of the translated human genes were mapped and/or localized in human tissues. Integration of transcriptomic and tissue microarrays data recently led to the completion of a first draft of a tissue-based map of the human proteome.[42] The map provides spatial information for proteins in 44 normal tissues, 20 human cancers and 46 cell lines. All images, data and information about application-specific antibody validation have been made publicly available.[43] The portal is organized in different builds based on protein functions and/or localization such as the subcellular, membrane, housekeeping, regulatory (e.g., transcription factors) and drugable proteomes as well as the secretome.

In the landscape of scientific organizations fostering proteomic research, HPA represents a standalone resource providing data and reagents to further investigate the human proteome based on images and expression pattern found in organs, tissues and cells. This enables the community to check for their proteins of interest, generate and suggest novel hypothesis to be tested, and provides one possible, independent source to support discoveries made with other methods.[44] The antibody-based map is complementary to recently established MS-based maps of the human proteome.[39,40] Among these are the Human Proteome Map [45] and the ProteomicsDB.[46] These two databases of MS data provide a web interface to search the proteomes of human cells, tissues and body fluids. They assemble the unique bioinformatics resource from existing databases involved in the Human Proteome Project [48] such as the PeptideAtlas,[40] ProteomeXchange [49] and Pride.[50] This is furthermore complemented with in-house experimental data. Currently, ProteomicsDB holds protein evidence for 93% of the 19,629 annotated human genes in Swiss-Prot and 22% of Uniprot, which includes isoforms, for a total of 86,771 entries.[39] ProteomicsDB and HPA provide independent evidence for 15,156 proteins and in their content overlap by 93%.

### 1.3 Aspects of translational proteomics: from sample profiling to clinical application

#### 1.3.1 Multiplex antibody arrays for biomarker discovery

There are a large number of assays that exploit IC and immunodetection to measure protein levels in biological samples. This section provides a brief overview of different proteomic technologies to introduce commons terms and methodologies (Figure 1).

On protein microarrays, a large number of miniaturized experiments are performed in parallel, using only small amounts of samples and reagents. This makes this technology perfectly suited for preclinical studies, where screening of large patient cohorts is required and the quantity of sample material is often limited. Protein microarrays can be classified based on the solid support used for protein or affinity reagent immobilization: planar microarrays use functionalized glass slides for the attachment of affinity reagents on distinct microspots, while bead-based microarrays use microspheres as solid support. Within planar arrays there are forward-phase affinity arrays where antibodies or other capture reagents are immobilized onto solid array surfaces and incubated with complex samples for multiplex detection of hundreds or thousands of proteins. Proteins captured on the array surface are typically detected via fluorescent labels, but there are a growing number of alternative read-out systems emerging for sensitive detection, including label-free systems.[51] Another format of planar array is the reverse phase protein arrays (RPPAs), where samples are spotted directly on a slide, allowing a higher sample throughput when compared to analyzing one sample at a time. The reverse arrays are a good choice for rapid screening and validation of candidate biomarkers in tissue and blood samples. In bead based microarrays also known as Suspension Bead Array (SBA) antibodies are covalently coupled to color-coded magnetic beads. Arrays are generated by mixing beads labeled with different fluorescent IDs and protein samples are functionalized with biotin prior to incubation with the array of beads. Antibody–protein binding event is revealed by the use of a fluorescent reporter functionalized with streptavidin (streptavidin–phycoerythrin [SAPE]) while a flow cytometric system which features a dual laser system is used to detect and record bead/antibody ID and SAPE intensity. The bead array setup offers several advantages, such as improved kinetics, higher flexibility, possibilities for automated washing steps and greater sample throughput as compared to planar arrays. For example, the xMAP® bead array technology (Luminex Corp., Austin, TX, USA) currently offers up to 500 parallel measurements in a single run.[52]

Protein arrays, or generally any type of immunoassay, can be also classified as single- or dual-binder assays depending on the number of antibodies that bind to an intended target in that assay. An improved selectivity and sensitivity (pg/ml range) are provided by dual-binder assays, as signal generation requires the binding of two separate antibodies to a common target protein. Dual-binder assays are the proximity ligation assay [53] based on DNA amplification, and the most common sandwich immunoassay (SIA), which is often mistakenly summarized as ELISA assays. Forward-phase affinity array format applying a labeled antibody for detection is indeed another example of a sandwich assay. Dual-binder assays development, however, is time-consuming because of the extensive optimization that is needed to identify compatible antibody pairs. In addition, SIAs do not allow the same degree of multiplexing (commercial kits currently offer up to 50-plex assays) because of the cross-reactivity between detection antibodies that often occur when combined in a mixture. As a consequence, single-binder assays are more commonly employed for discovery-oriented studies and SIAs for the validation of selected targets in extended sample sets.

#### 1.3.2 Clinical applications: traditional and advanced immunoassays

Over the past 40 years, ELISA has been one of the analytical methods most extensively used for clinical measurements. SIA ELISA can be used for semi- or absolute quantification if standard curves are applied.[54] ELISA is considered a highly specific, sensitive, as well as very standardized and reliable method for clinical applications, but it shows some disadvantages such as the requirement of relatively large sample material, being a dual-binder assay as mentioned above, low level of multiplexing and the need for a working pair of antibodies. Novel methods, based on ELISA to some extent, have emerged overcome the limitations of the standard ELISA method.

Fredriksson *et al*. described a novel multiplexed sandwich ELISA method using two specific antibodies linked to DNA fragments. Once the antibodies are bound to the analyte, the DNA proximity ligation occurs and the signal is amplified by PCR. This methodology, apart from increasing multiplexity, avoids washes and the use of fluorescence.[53] To meet the need of functional affinity reagents pairs, alternatives to antibodies have been tested. Gold *et al*. described an aptamer-based multiplexed proteomic technology. DNA-based aptamers containing chemically modified nucleotides, that mimic amino acid side chains, function as protein-binding reagents.[55]

Moreover, a number of alternative read-out systems are emerging that aim to provide a more sensitive detection, including label-free systems based on label-free biosensors. Immunosensors combine nanoparticle-based solid surfaces where immunochemical reactions occur, coupled to a transducer to quantify the signal. According to the type of transducer system, immunosensors can be classified as electrochemical, optical, piezoelectric, magnetic or thermometric.[56] Among them, electrochemical immunosensors are the most broadly used for quantitative analysis of clinical biomarkers.[57–60] Their main innovation resides in the introduction of nanomaterials in their design (i.e., gold nanoparticle, carbon nanomaterial, quantum dots, etc.), that significantly increase the solid surface area for affinity reagents contributing to high sensitivity performance.[61] Therefore, immunosensors may soon provide convincing advantages over standard immunoassays, including miniaturized and renewal devices, sensitivity, rapid detection, degree of multiplexing, cost [62] and automation.[57] Important parameters for clinical laboratories will, however, remain in the performance characteristics such as precision, robustness, reproducibility and sustainability of these devices. Consequently, more work needs to be dedicated to meet clinical requirements before novel immunosensors can become a platform within clinical diagnostics, in particular for the bedside as point of care systems. Current and emerging immunoassay systems are described in Table 2.

**Table 2.** **Analytical features of current and emerging immunoassay systems**.

	Affinity reagents	Surface	Quantification	Multiplex (affinity reagents)	Sample volume	Dynamic range (logs)	Reference
ELISA	Abs	Microplates	Fluorescence or Chemiluminescence	Limited	>25 µl	2–3	[54]
Mesoscale	Abs	Microplates	Electrochemiluminescence	Limited	>25 µl	2–5	[63]
Singulex-Erenna®	Abs	Magnetic microparticles	Fluorescence (Ab-biotinylated)	No	10–100 µl	3–4	[64]
Bead based Sandwich assay	Abs	Magnetic microparticles	Fluorescence (Ab-biotinylated)	Scalable	50–100 µl	3–4	[65]
Bead based Antibody array	Abs or antigens	Magnetic microparticles	Fluorescence (Streptavidin-labeled)	Scalable	3 µl	3–4	[52]
Olink-Proximity Ligation Assays	Abs	In solution	PCR amplification	Scalable	1 µl	2–5	[53]
Quanterix-Simoa ®	Abs	Magnetic beads	Fluorescence (Streptavidin-labeled)	Scalable	150 µl	2–4	[66]
Somalogic- SOMAmer®	Modified Aptamers	Magnetic beads	DNA microarrays or qPCR	Scalable	150 µl	7–8	[55]
Simplex	Abs	Glass nanoreactors	Fluorescence (Ab-biotinylated)	Limited	<25	3–4	[67]
Immunosensors	Abs or aptamers	Nanomaterials	Chemiluminescence or label-free	Scalable	10 µl	3–7	[60]

#### 1.3.3 Requirements for experimental design and data analysis

To provide a critical perspective on the role and future of IC technologies, here we provide a brief discussion on the current status of proteomics associated clinical and preclinical studies. Protein profiling of biological specimens by antibody assays or MS-based approaches has enabled a massive expansion of the field of biomarker discovery. This has led to a large number of scientific publications about the matter; however, very few of these discoveries have been applied in a clinical setting.[9,68] This huge discrepancy between the number of postulated novel candidate biomarkers and those introduced in the clinic highlights the challenges posed by the long and difficult path of biomarker validation.[69] In the past, a lot of effort has been put into developing novel, high throughput (number of samples) and multiplexed (number of targets) technologies with increased sensitivity. This was based on the assumption that the main reason for the failure in finding novel relevant biomarkers was limited sensitivity and narrow dynamic ranges. However, there was also a lack of attention to study design, interindividual heterogeneity, biological meaning of the discovery, as well as assessment of analytical performance of assay and technology.

As we discussed previously (Section 1.1), the specificity of an antibody applied in any immunoassay cannot be taken for granted, even when we are talking about a commercial product. One emblematic case of false discovery covers the case of CUB and zona pellucida-like domains protein 1 (CUZD1). CUZD1 is a highly pancreas-specific protein hypothesized to be a potential marker for pancreatic ductal adenocarcinoma in circulation. Using a commercial assay, levels of CUZD1 measured in a cohort of 100 serum samples confirmed it as a candidate biomarker. Nevertheless, further investigations to assess the specificity of the assay revealed that CUZD1 was instead the well-known cancer biomarker cancer antigen 125.[70]

Moving to discuss the aspect of study design, the identification of a candidate protein as a biomarker for a disease is often pursued using case–control studies.[71] This approach, which is the most common in epidemiological studies of proteomics compares protein levels between multiple groups divided by disease status and aims to discover proteins with differential “expression”. At the stage of sample recruitment, there are several important considerations to make: to establish a large enough sample size to achieve a sufficient statistical power that allows us to detect small difference between groups,[72] to minimize preanalytical variables (e.g., differential handling of the samples) [73] in order to enable conclusive interpretations related to the phenotype rather than the sample type and sample handling [73,74] and to properly match samples in order to measure disease-related difference rather than individual heterogeneity.[72] For the recruited samples, analysis should be performed after randomization, preventing possible association of experimental factors (e.g., sample collection order, reading order) with protein profiles [72] and should be accomplished during sample collection to avoid confounding by admission order. Finally acquired proteomics data are often subject to normalization so as to minimize the variability introduced by technical factors, for example, the differences in sample loadings, concentrations, or variation between plates or batches.

In this perspective, the advantage of high-throughput affinity-based technologies as compared to MS, the workhorse in proteomics, is therefore to measure a large number of samples required for a meaningful statistical analysis in a limited amount of time. For this reason, despite the discussed issues of antibody specificity, affinity-based approaches still have a critical role in basic and translational proteomics.

#### 1.3.4 Affinity based bio-fluids profiling

When the content of cellular proteins changes in relation to disease, it is preferable to measure those changes via analysis of accessible body fluids rather than affected tissue. Because of minimal invasiveness and ease of collection, body fluids such as blood, urine, saliva or even sweat represent appealing sources for a direct diagnosis and patients monitoring. Among these, blood plasma represents a systemic view of the current health status and is certainly the most commonly used sample type due to routine collection.

SBA format has been utilized for plasma biomarker discovery within various disease contexts.[44,75–77] For example, Bachmann *et al*. [78] identified muscle-specific proteins as candidate markers for cerebral malaria by screening more than 1,000 proteins in 700 plasma samples from malaria-infected children and controls. Byström *et al*. identified proteins in relation to multiple sclerosis [76] and Ayoglu *et al*. in studying muscular dystrophies.[79] In other recent works, bead-based sandwich ELISA assays were utilized to validate previously identified plasma protein candidates in more than 1,000 patient samples, within both AD [80] and prostate cancer.[81]

RPPA format is a good choice for rapid screening and validation of candidate biomarkers in blood samples. Grote *et al*., for example, profiled the level of CA19-9 in pancreatic cancer.[82] Values compared with a standard ELISA showed a correlation of 75–95% and comparable area under the curve values and specificity. Nevertheless, analysis of serum and plasma samples spotted on slides limits applications to higher abundant proteins, as demonstrated by Janzi *et al*. for immunodeficiency.[83,84] To improve sensitivity, to lower cross-reactivity and to overcome the complexity and high dynamic range of the samples, it would be beneficial to adjust sample composition. Longo *et al*. have, for this purpose, coupled such assays to hydrogel nanoparticle enrichment for the discovery of biomarkers of melanoma in serum.[85]

An interesting planar array format called the antibody colocalization microarray [86] has been recently applied to quantify proteins in 15 breast cancer patients and 11 healthy controls. Antibody colocalization microarray is a dual capture assay, in which detection antibody is spotted and localized with its matched capture antibody, which results in an array of nano-sandwich assays. The “spot” format allows one to multiplex the immunoassay, overcoming the problem of cross-reactivity and reaching a sensitivity in the low pg/ml. Pla-roca *et al*. measured in their study 50 proteins and compared these with to conventional multiplex assays with low multiplexing. They identified and measured six known biomarkers obtaining concordant results with previous studies.[86]

Profiling of protein glycosylation in serum and plasma with lectins or glycan-binding antibodies and autoantibody characterization and quantification also has been implemented on affinity-based microarray platforms.

Moreover, microarrays have been applied for quantitative serological analysis within pregnancy screening for IgG and IgM against cytomegalovirus, herpes simplex virus, varicella zoster virus, *Chlamydia trachomatis* and others.[87] Other clinical applications for planar arrays are the detection of pathogen antigens and antibody response in infections and allergies. In particular, ImmunoCAP® ISAC by Phadia (now Thermo Scientific) is already available in the market for clinical testing of more than 100 allergens. Other antigen arrays have been developed to detect autoantibodies in several different diseases such as celiac disease,[88] multiple sclerosis [89] and cardiomyopathies.[90]

Proximal fluids on the other hand are less ideal for large-scale clinical screening or monitoring procedures due to more cumbersome collection procedures. Nevertheless, these samples represent a valuable source of biomarkers because they are in direct contact with affected tissues. Consequently, these body fluids may contain higher levels of disease-related proteins of cellular or micro-environmental origin compared to systemic fluids. Such proximal fluids include synovial fluid in the context of rheumatic disease, urine for kidney or metabolic disorders, saliva for assessment of periodontal disease and cerebrospinal fluid (CSF) for neurological disorders. Despite the potential for discovery of proteins reflecting disease pathogenesis in proximal fluids, affinity-based studies of protein profiles are still limited with the majority of published studies based on CSF analysis. Oláh and colleagues recently reported analysis of CSF on commercially available planar microarrays with the aim to find proteins related to AD.[91] The array contained 653 antibodies and protein detection was mediated through sample biotinylation and fluorescent read-out. The study included CSF from 25 patients and 25 controls that were pooled into one AD and one control pool prior to analysis. By comparing the levels in the two pools, the authors reported seven proteins with disease-associated levels. Verification of the observed profiles with Western blot could, however, not confirm either the presence or altered levels of the selected proteins. A similar approach using antibodies and direct labeling of proteins has also been applied within multiple sclerosis, as reported by Häggmark *et al*.[92] They used a bead-based microarray system to analyze the levels of 43 proteins in CSF from 339 individuals, reporting two proteins to be associated to inflammation and disease progression. For both proteins, several antibodies were used in parallel, revealing highly correlating results and for one of these proteins, a sandwich assay was set up that also resulted in concordant observations. The levels of these proteins were then validated in a second sample collection with 234 additional CSF samples.[76] In addition to CSF, affinity proteomic strategies have been utilized for analysis of saliva, where Fine and coworkers performed multiplex cytokine profiling using a bead-based microarray system in sandwich ELISA format.[93] Out of 21 proteins analyzed, the levels of two proteins were found related to bone loss in periodontal disease.

#### 1.3.5 Affinity-based cell and tissue protein profiling

While proteins in body fluids are, to a large proportion, the result of secretion and leakage processes from different organs and tissues of the body, tissue analysis allows observing directly the status of the affected cells. In particular, the study of post-translational modifications (PTM), protein–protein and kinase–substrate interaction involved in signal transduction cascade gives the possibility to further characterize the activation status of pathways that drive cancer genesis and progression. One of the main issues to face when studying tissues is the complexity, due to a mixture of several different cellular types that sustain each other by a fine network of paracrine signals.[94] Disassembling this structure is the basis for characterizing a pathway status in cancer onset and progression, and is achieved via laser capture microdissection [95–97] or cell sorting.[98] The small amount of available tissue, that becomes even smaller when looking at cell subpopulations only, makes it necessary to use miniaturized and sensitive techniques such as RPPA.[99] RPPA found several applications in bench-to-bedside research, providing new insights in tumor signaling architecture. When working with few small biopsies, a major issue arises from whether or not its expression profile is representative for the whole tumor.[100] Recent data suggest that multibiopsies may be more representative than single.[101] RPPA also helped in revealing the differences in the signaling network of patient-matched primary and metastatic cancer lesions.[102–104] These findings can be partially explained by the influence of the different microenvironments surrounding primary tumor and metastasis and suggests that patients with multiple metastases should be treated using a combination of different target therapies selected considering tumor-specific signaling as well as tailoring stromal markers.[105] Beside solid tumors, RPPA has been applied also for studying leukemia revealing differences in protein expression and activation in cellular subpopulations,[98] identifying potential biomarkers [106] and drug targets.[107] Due to the encouraging data, RPPA has been applied to several clinical trials for the evaluation of single and combinatorial drug efficacy in patients with metastatic and locally advanced cancers.[108–113]

An indirect approach to analyze a disease at a tissue level exists by investigating cells that secede the primary tumor and intravasate into the circulation. These cells are also known as circulating tumor cells (CTCs). These CTCs have been studied for their ability to form metastases at distant sites [114] and hundreds of clinical trials are evaluating of CTCs as cancer biomarkers for diagnosis, prognosis, prediction, patients stratification and response to therapy.[115]

The methods aiming at the use of CTCs of biomarkers are based on purification, counts and characterization of cells in the bloodstream. Isolation of CTCs is a major challenge since they represent approximately one cell each few millions of blood cells.[116] The antibodies used to capture and characterize CTCs may target epithelial proteins such as Epithelial cell adhesion molecule, cytokeratines, and mesenchymal markers (N-cadherin and vimentin). A negative control antibody for CD45 (a leukocyte marker) and nuclear staining are used to discriminate between blood cells, circulating benign epithelial and actual tumor cells. Improved assay specificity is obtained when antibodies for tissue-specific and tumor-specific markers are included in the analysis both as capturing or staining reagents. CellSearch is currently the only US Food and Drug Administration-cleared technology for CTC analysis.[115] CTC counts have been monitored in patients with metastatic breast, colorectal and prostate cancer showing to be predictive of overall survival.[115] To improve the capture efficiency and purity of CTCs, antibody-based strategies have been coupled to microfluidic devices. A geometrically enhanced differential immunocapture (GEDI) microfluidic device have been applied to count and characterize CTCs in prostate cancer patients.[117] The system applies a very specific capture using an antibody that binds a prostate-specific membrane antigen, a marker highly expressed by malignant prostate epithelial cells, and a novel 3D geometry based on size separation minimize the contaminations of leukocytes. The GEDI device showed a 2–400 fold higher sensitivity than CellSearch in capturing CTCs.[117] A similar system was developed and applied to study circulating pancreatic cells and pancreatic CTCs. Here, a GEDI device was implemented with method for chemical extraction of whole nuclei to allow a more accurate genetic analysis of circulating cells providing a better picture of disease progression.[118] Investigation and application of CTCs as biomarkers is still at an early phase. The heterogeneity observed in CTCs that reflects tumor heterogeneity (intra- and interorgans) will imply the need of tumor-specific assays that can lead to an accurate characterization of different cellular clones and standardization and clinical validation of novel methods.

### 1.4 Linking MS with antibody-based assays

Strategies for biomarker discoveries based on antibodies have several positive features some of which are outlined below:

- Antibody assays are often hosted in microtiter plates or on reverse-phase microarrays offering high sample throughput to better meet statistical requirements;
- Immunoassays require minimal amount of sample offer reproducibility, are compatible with existing analytical expertise and equipment and can be analyzed in a time suited for a clinical laboratory;
- Capturing targets of interest from different biological samples is possible and generally requires minimal sample preprocessing;
- Capturing targets of interest can increase the sensitivity of the assay;
- Developing clinical assays from an antibody-based discovery makes use of already selected epitopes and affinity reagents to build and translate assays into the intended final format.

In contrast to this, there is an increasing awareness of antibodies being prone to cross-react and display off-target binding.[119] In particular for single-binder assays, this calls for highly selective and thoroughly validated antibodies, including the antibodies be tested before being applied (e.g., using orthogonal methods) and evaluated with the samples in the system chosen for the final analysis. One option is to use multiple antibodies for the same target, building paired and dual-binder assays, or to apply other modes of detection that allow for the determination of which components contributed to discoveries. The latter clearly points toward exploiting MS strategies as an important complement to affinity-based proteomics and the combination of the two involves actually a two-way advantage.

MS proteomics indeed has been for years the leading force for non-hypothesis-driven biomarker discovery and investigation of therapeutic targets. Nevertheless, some of its limitation is particularly significant in the context of translational proteomics. An unsurpassed specificity of protein identification together with PTM and isoform characterization is contraposed to limited sensitivity for low abundant proteins, which are believed to be the most interesting source of biomarkers. Low dynamic range and matrix effects in complex samples are the main instrumental drawbacks leading to limited deepness of analysis of complex biological samples such as tissues and plasma, characterized by dynamic range, respectively, of 10^6^ and 10^10^. To increase deepness of analysis, a longer chromatography separation may be applied or more extensive sample preprocessing and fractionation, with the effect of lowering high throughput and reproducibility. Recent development of IC strategies in combination with MS techniques have made it possible to overcome some of the technical barriers often associated with MS. Antibody enrichments have been applied to enhance the analysis of rare classes of proteins such as phosphopeptides,[120,121] glycoproteins,[122] or protein ubiquitination [123] in shotgun discovery pipelines; to improve sensitivity for quantification of low abundance proteins; to study protein–protein interactions.[124–127] In the following sections, we will describe some integrated strategies that may strongly impact the future of translational proteomics with a special focus on IC-MS applied to development of novel clinical assays.

#### 1.4.1 Development of IC-selected reaction monitoring-based clinical assays

A very promising analytical method comes from the coupling of immunoenrichment with targeted MS approaches. Such MS approaches gained popularity in recent years under the names of selected reaction monitoring (SRM) and parallel reaction monitoring.[128] SRM, also known as multiple reaction monitoring, describes a robust MS technique used for measurements, quantification and detection. Particularly appreciated for its analytical performances, it has been elected method of the year 2012 by Nature Methods.[129,130] Accuracy, high specificity to discriminate different proteoforms PTM or isoforms, reproducibility [131,132] and sensitivity together with the possibility to perform multiplexed analysis [133–135] make the SRM concept a valid future alternative to standard clinical assays. Abatiello and colleagues performed recently a large study of reproducibility which involved 11 laboratories. A hundred and twenty five peptides derived from 27 cancer-relevant proteins were measured with standardized that applies a stringent a quality control system, called system suitability protocol. Intra- and interlaboratory reproducibility was <20% with a sensitivity of subnanogram/ml level in depleted plasma.[132] While such assays have been demonstrated to be robust and reproducible with good applicability in preclinical assays, current sensitivity would limit their analysis to higher abundant analytes of clinical interest.

#### 1.4.2 IC of peptides for MS

Immunoaffinity enrichment based on antipeptide antibodies coupled to stable isotope standards and capture by antipeptide antibodies (SISCAPA) was introduced by Anderson in 2004.[136] Antibodies raised toward proteotypic peptides are hosted on solid support and incubated with digested samples that were spiked with known amounts of corresponding heavy isotope-labeled peptide standards. Enriched heavy and light peptides are eluted from the solid support in a small volume that is less complex and more compatible for downstream SRM analysis. It was demonstrated that IC-SRM assays reach limit of detection (LOD) of ng/ml, which can even be decreased to pg/ml when higher volumes of starting plasma could be applied. The method has good precision (median coefficient of variation, 12.6%) and intralaboratory reproducibility.[137,138] Whiteaker *et al*. have developed a multiplexed method including 220 antipeptide antibodies to detect 89 proteins in human plasma reaching down to concentrations of 0.5 fmol/µl. This effort has demonstrated the feasibility for a single laboratory to develop assays for a large number of proteins.[139] The first example of IC-SRM assay was provided by two cardiovascular biomarker interleukin 33 (IL33) and troponin I (cTnI). These proteins were measured in plasma samples with a linear analytical range of 5,000–1.5 ng/ml. Limit of quantification (LOQ) for both proteins were 2.8 (cTnl) and 4.7 (IL33) ng/ml and a correlation of (R = 0.89) with the commercial assay was reported.[140] Assays based on antipeptide antibodies and postdigest enrichment will help minimize interference arising from endogenous antibodies, as these otherwise have the potential to cross-react with binding reagents of classical immunoassays (e.g., detection antibodies). Hoonfnagle *et al*. [141] applied immuneaffinity peptide enrichment coupled with SRM to quantify thyroglobulin (Tg) in serum. Tg is one validated serum biomarker for thyroid carcinoma and its levels are monitored during treatment of thyroid cancers. Immunoassay tests used for quantification of Tg are, however, affected by anti-Tg antibodies highly abundant in patients with thyroid carcinoma, which can result in underestimation of level of Tg in serum. Tg was immune-enriched from 100 µl of digested serum and was detected down to a level of 2.6 ng/ml (4 pmol/l) revealing good correlation (rho > 0.8) with immunoassay test. Moreover as peptide enrichment is very efficient, it is possible to bypass separation by liquid chromatography as peptides from enriched plasma samples can be analyzed within seconds using MALDI-MS.[141,142,143] MALDI-MS coupled to automated plate loading enables the analysis of up to 24 plates a day containing 2400 digested samples,[144] opening up such assays for diagnostic applications. Alternatives to specific enrichment of one specific peptide is selective depletion of peptides using antipeptide antibodies generated toward a common amino acid motif present in many proteotypic peptides.[145] Furlong *et al*. have instead shown that universal peptide motifs can be used for the enrichment of several different targets.[146] Other initiatives such as the HPA have demonstrated that polyclonal antibody repertoire, generated towards protein fragments, can be used for peptide enrichment together with spike in of heavy labeled protein fragment standards.[147,148]

#### 1.4.3 IC of proteins for MS

Alternatively to peptide enrichment, IC of intact proteins is possible and presents some noteworthy advantages: It allows for the increase in sample volume (e.g., plasma) avoiding the cost and performance issues related to the digestion of large volumes, it further increases the sensitivity of the assays to LODs of pg/ml and bypasses the need to develop of antibodies against prototypic peptides. An IC-SRM assay was developed for the detection of the variant of human chorionic gonadotropin (hCG) in serum and urine samples and validated according to the guidelines of the European Medicine Agency for precision accuracy and linearity (LOD: 2 IU/l in serum and 5 IU/l in urine). Human CG is a hormone produced by the placenta, thus tested for pregnancy detection but also secreted by nontrophoblastic cancers such as seminoma, and islet cell tumor. Today hCG is also used as a doping agent, and it constitutes a protein that has been described in several variants, which each can stem from different origins and have different biological effects. A monoclonal antibody was developed with the selectivity to enrich seven known variants of hCG. At present, variants of human growth hormones were measured in pregnant women, patients with testicular cancer and a man who was administered with Pregnyl, as pharmaceutical hCG.[149,150] Using a similar approach, isoforms of cancer biomarker progastrin-releasing peptide were profiled in the serum of patients suffering from small cell lung carcinoma, non-small cell lung carcinoma and medullary thyroid cancer patients. Ratios of progastrin-releasing peptide isoforms in the three cancers were also determined and found to be different.[151,152] IC-SRM assays were compared with automated time-resolved immunofluorimetric assays showing good correlation; however, the values determined by the MS method values were systematically lower by 30%. IC-SRM assay based on protein enrichment have also been developed to discriminate active forms of the parathyroid hormone in patients with hyperparathyroidism [16] and different forms of neuron-specific enolase in serum (LOD = 11 pg/ml; LOQ = 38 pg/ml).[153] Data independent acquisition (DIA) methods like sequential window acquisition of all theoretical fragment-ion spectra (SWATH) analysis have even higher throughput in number of targets per sample, and constantly growing data repositories allow decoding and identification of more than 10,000 proteins.[154] Also, SWATH analysis of plasma has been shown to have LOQ down to 5 ng/ml in depleted plasma.[155] Additionally, DIA analyses have the advantage of storing the data as digital maps, which allows reanalyzing of the data at a later stage if needed. DIA methods will further improve their analytical power taking advantage of affinity enrichment steps. For example, solid phase extraction of glycopeptides has been applied to enrich and profile glycoprotein in prostate cancer tissues.[156]

#### 1.4.4 Antibody and target validation by MS

Recently, IC experiments followed by MS analysis have been used as complementary methods to classical immunoassays, such as antibody bead arrays. In this single antibody assay setting, splitting the bead population after sample incubation for different read-out methods corroborated differential abundance of plasma proteins. For the tested complement proteins, eluates from the enrichments were applied to MS (and Western blot), showing concordant results to flow cytometer read-out.[157] In such MS experiments, the antibody becomes not just a tool for enrichment but a reagent to be tested for quality. Indeed, the previously described SBA method is not only a method to discover biomarkers but also a platform that allows to efficiently identify affinity reagents suitable for body fluid analysis and for the subsequent development of sandwich assay. It is known that SIAs require evaluating different reagents and their use in a specific sample. Knowing if antibodies cross-react or bind to an off-target will, therefore, help to select those binders with the most suitable enrichment capabilities for the sample of interest. It has been shown that a preliminary IC-MS-based screening of antibodies providing relevant differential profiling allows the selection of only promising candidate biomarkers and specific reagents to the following step of validation.[81] Marcon *et al*. recently published a workflow based on IC-MS scoring antibody performances in immunoprecipitation. There, more than a thousand immunoprecipitation assays using recombinant antibodies were performed, and the antibodies enriching their target were defined as ‘IP Golden standard’ in tissue lysates.[158] Korbakis *et al*. developed an approach based on IC-SRM to evaluate the best monoclonal antibodies. They aimed to build a sensitive ELISA to measure TEX101 a marker for male infertility in seminal plasma and serum,[159] thus evaluated the specificity of the generate monoclonal antibodies by analyzing the immunoprecipitated protein using SRM.[160] In summary, IC-SRM assays have been demonstrated to have analytical performance, robustness and specificity that are suitable for biomarker and antibody validation. Such methods can serve as a bridge to build sandwich ELISA assays and upon further advancement may even serve as their alternative. This suggests that a growing number of studies will utilize MS as a validation tool to link between discoveries made on affinity array and clinical assays.

#### 1.4.5 Atomic mass spectrometric analysis to enhance cell profiling and molecular imaging

Flow cytometry coupled to cell sorting and IHC have had an important impact on preclinical research and clinical diagnostics. They are, however, both limited in the analysis of panels of molecules by the maximum numbers of antibodies applicable in the same analysis (usually between 5 and 10). Antibody detection is usually based on fluorescence or chromogenic reactions, and both are limited in multiplexing due to the number of noninterfering spectra fluorophores or of enzymes; difficulties in finding combinations of animal-specific secondary antibodies; the autofluorescence or a dense chromogenic deposit that is difficult to quantify and a reduced analytical dynamic range and spectral overlapping.

While the use of MS for absolute quantification of proteins and peptides captured by antibodies provides a noteworthy improvement in specificity, another promising application of MS in IC strategies is offered by the detection of antibodies labeled with rare elements by atomic MS, found in mass cytometry (MC).

In MC, cells are stained with antibodies tagged with isotopically pure transition elements and each antibody is labeled with different mass elements. Cells are then nebulized in single cell droplets and ionized in their atomic constituents by inductively coupled argon plasma. An atomic mass spectrometric analysis is then performed by a time-of-flight (TOF) instrument providing quantitative information. MC is not as sensitive as quantum-efficient dyes, but the lack of background signal (autofluorescence) can reduce sensitivity difference compared to fluorescent dyes.

Offering the opportunity to analyze simultaneously a high number of parameters (currently 50 with the possibility to extend up to 100), MC adds a great value to single-cell analysis.[161] MC has been mostly applied to profile markers on hematopoietic cells from peripheral blood and bone marrow, giving the possibility to study more details of the immune system and its role in a large variety of diseases and conditions such as autoimmune diseases,[162] injurious inflammatory processes,[163] infections [164] and cancer.[165]

Antibodies labeled with transition elements are applicable in a similar way for tissue staining to map molecular markers in tissue section.[166] Two main methods have been described for ionization and detection of labeled antibodies in tissue: scanning MC and multiplexed ion-beam imaging.[167] An antibody detection based on MS would strongly advance current methods for diagnosis, patient stratification and therapy decision based on tissue and cellular imaging which would benefit from the parallel detection of multiple biomarkers.

## 2. Expert commentary

Antibody-based assays, such as SIAs and IHC, have for years been the golden standard methods in clinical applications. Nevertheless, many methods based on antibody detection have been watched with criticism because of limited specificity, cross-reactivity and batch-to-batch variability. These factors may undermine the importance of reliability, reproducibility in future application for discovery. More stringent evaluation schemes of antibodies in the context of each application are required. Antibody applications will most certainly continue to advance the efforts in the search for novel biomarkers and to provide biological insight for a better understanding and treatment of diseases. Novel and existing affinity array formats are successfully demonstrating a growing reliability and thus translatability to clinical applications such as diagnostic and personalized medicine. We foresee that affinity reagents will become even important tools to enhance MS-based analysis, and vice versa, not only for target enrichment and the investigation of protein complexes but also as robust methods to assure quality antibody selectivity. This will make MS the bridging element toward quantitative clinical assays. Upon further improvements in terms of analytical sensitivity and speed, new models of clinical assays will emerge that start with an affinity capture and end with an MS read-out. As several studies have reported, IC-MS assays can be robust and perform as the current golden standards. MS can also serve as a complement method for putative biomarker validation, for example, if two paired antibodies are not available. Moreover, MS can be used to screen and verify antibody targets to support the generation of sandwich assays and thereby reduces time, cost and effort. This trend assisting a turning point and further development of antibody-based strategies will continue to exploit alternative regents other than those derived from animal immunization.

## 3. Five-year view

Translational aspects of proteomics have been going through a period of stall due the lacking success stories that introduce new biomarkers to patient care. The disappointment, partly due to high expectations and massive investments, is not just related to antibody quality or lack in MS sensitivity, but also due to how proteomics has failed to design biomarker studies.[168] In general, all the high performance proteomic studies that exploited technology advancements, the multiplexing and high-throughput capabilities, produced a large amount of data, novel biological information and candidate markers of disease that have the potential to be translated to clinical applications. This can be successful, however, only if clinicians, biostatisticians and experimentalists design the studies of new discoveries and validation together. In the coming years, the path from initial discoveries to a first testing in a clinical setting should be shortened and allow a first assessment of every candidate. Clinical laboratories are therefore important partners for proteomic scientist to collaborate with, as these set the standards on how patient material should be analyzed and how accurate, sustainable and reliable the data need to be. In the next few years, we may observe a turning point, if cross-platform validation and continued integration of analytical and bioinformatics tools can serve to streamline the transition from discovery to validation and help to avoid over-fitting of the data. The increasing number of biobank initiatives, which aim to provide high-quality and standardized specimen for preclinical studies will foster accuracy in biomarker studies and will implement longitudinal investigations. Moreover, stringent requirement will be posed for data generation, interpretation and validation studies. For antibody-based strategies, the key will be in putting together even a greater effort to validate high-quality reagents and evaluate alternative sources of affinity reagents. It will become more evident that antibodies must be assessed directly in the sample context and application preferably while being compared with other methods and reagents. It will also become obvious that in a complex biological solution, antibodies may not always capture only one single protein but may bind several different proteins with respective interaction affinities. Given that the target of interest is present and that appropriate read-out, such as dual-binder assays or MS, are used, a particular proportion of the generated data should contain information of the selectively captured and targeted protein. In the long run, antibodies should always be judged based on their application and context-dependent selectivity rather than the anticipated specificity.

**Key issues**

- Performance of mass spectrometry instrumentation will have to be further improved in terms of speed, sensitivity and sample requirement.
- Strategies to fuse affinity- and mass spectrometry-based assays will be driven by clinical needs.
- Clinical labs need to guide proteomics on how future assays could look like.
- Clinicians, biostatisticians and experimentalists need to improve study designs of proteomics efforts to enable that potential discoveries survive validation into clinical utility.
- More antibodies and alternative binders will become available that need to be validated in the intended application.
- Antibody performance is context- and application-dependent and needs to be assessed.

## Financial & competing interests disclosure

*The authors have no relevant affiliations or financial involvement with any organization or entity with a financial interest in or financial conflict with the subject matter or materials discussed in the manuscript. This includes employment, consultancies, honoraria, stock ownership or options, expert testimony, grants or patents received or pending, or royalties*.

## ORCID

*Jochen M Schwenk* 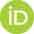 http://orcid.org/0000-0001-8141-8449
